# Post-Decision Wagering Affects Metacognitive Awareness of Emotional Stimuli: An Event Related Potential Study

**DOI:** 10.1371/journal.pone.0159516

**Published:** 2016-08-04

**Authors:** Michał Wierzchoń, Eligiusz Wronka, Borysław Paulewicz, Remigiusz Szczepanowski

**Affiliations:** 1 Consciousness Lab, Institute of Psychology, Jagiellonian University, Krakow, Poland; 2 Psychophysiology Lab, Institute of Psychology, Jagiellonian University, Krakow, Poland; 3 SWPS University of Social Science and Humanities, Faculty in Katowice, Poland; 4 SWPS University of Social Science and Humanities, Faculty in Wroclaw, Poland; Vrije Universiteit Brussel, BELGIUM

## Abstract

The present research investigated metacognitive awareness of emotional stimuli and its psychophysiological correlates. We used a backward masking task presenting participants with fearful or neutral faces. We asked participants for face discrimination and then probed their metacognitive awareness with confidence rating (CR) and post-decision wagering (PDW) scales. We also analysed psychophysiological correlates of awareness with event-related potential (ERP) components: P1, N170, early posterior negativity (EPN), and P3. We have not observed any differences between PDW and CR conditions in the emotion identification task. However, the "aware" ratings were associated with increased accuracy performance. This effect was more pronounced in PDW, especially for fearful faces, suggesting that emotional stimuli awareness may be enhanced by monetary incentives. EEG analysis showed larger N170, EPN and P3 amplitudes in aware compared to unaware trials. It also appeared that both EPN and P3 ERP components were more pronounced in the PDW condition, especially when emotional faces were presented. Taken together, our ERP findings suggest that metacognitive awareness of emotional stimuli depends on the effectiveness of both early and late visual information processing. Our study also indicates that awareness of emotional stimuli can be enhanced by the motivation induced by wagering.

## Introduction

Recent years have brought a vast body of empirical studies and theoretical models investigating neural correlates of consciousness (NCC, see e.g. [1–4] for the review). Most research has focused on the identification of either the brain structures or brain networks responsible for conscious processing (see e.g. [5–6]). This research programme is also supported by EEG studies that allow a more finely tuned analysis of the time course of brain activity evoked by conscious perception and observed with neuroimaging technics (see. e.g. [7]). Recently, a vivid discussion on electrophysiological NCCs has also arisen (see: [8–9] for the review). The debate mainly concerns the time window in which the signatures of conscious processing should be observed with event-related potential (ERP) components. Several studies have attempted to investigate what the earliest psychophysiological neural correlates of consciousness are (see e.g. [10]). Some researchers agree that visual awareness coincides with differences in the early stages of sensory processing which is reflected by more pronounced amplitudes of P1 [7]. In addition, several studies suggest that visual awareness is reflected by general enhanced negativity in the N1-N2 range (so called visual awareness negativity, VAN. See e.g. [8, 11–12] for the review). On the other hand, many researchers disagree that awareness is related to enhanced early visual processing components and suggest that the electrophysiological neural correlate of consciousness is related to post-perceptual processing. Therefore, it has been claimed that differences in visual processing between seen and unseen stimuli should instead be observed with a P3 component (see e.g. [13–14]).

Importantly, the outcome of this discussion seems to be significant from a theoretical point of view as it adds to the knowledge on the time frame of awareness, i.e. it allows us to decide whether consciousness is rooted in early visual processing or whether late visual processing and attention are necessarily involved. It seems that the presence of early ERP correlates of visual awareness corroborate approaches that associate visual awareness with differences at early stages of visual processing (see e.g. [3]). On the other hand, the studies indicating later psychophysiological correlates contribute to alternative theories which postulate that awareness is more likely to be associated with later stages of information processing, i.e. with attention [2] and metacognition [15]. The present research aimed to investigate electrophysiological NCCs with complex stimuli that have well-defined and distinct ERP correlates responsible for stimulus feature identification, specifically with facial emotion expressions.

Facial emotion expressions seem to be a good candidate for investigating early processes that may contribute to awareness. This is because the ERP correlates of face detection and facial expressions identification have been extensively investigated and described elsewhere [16–17]. Several researchers suggest [18–20] that at least two ERP components should be closely inspected: N170 (increased ERP negativity 130–200ms after stimulus onset) and early posterior negativity (EPN, a negative ERP potential observed for a time window of 150-350ms after stimulus onset). N170 displays right-hemisphere lateralisation, and is usually recorded over the occipito-temporal cortex (which is consistent with the fusiform gyrus and inferial-temporal gyrus location [21–25]. This component is related to structural encoding of faces and is consistent with the function associated with the structures generating it [25]. EPN is also usually observed over the occipito-temporal cortex, but it has been linked to selection of visual stimuli that have affective and motivational significance [26] such as facial emotion expressions. Findings from recent ERP and neuroimaging studies indicate that the EPN component reflects widespread activation of the occipito-temporal brain areas engaged in visual processing [27–29]. It is also worth noting that both N170 and EPN are early visual components that can also be observed under subliminal presentation of facial expressions [18–19, 30]. However, it is interesting to see whether the amplitudes of these ERP components vary to some extent under aware and unaware conditions. This is especially interesting as these components are usually observed in the time window in which the previously mentioned VAN component is usually recorded. This is also important as the VAN time window is not clearly determined (i.e. VAN is usually observed around 200ms after stimulus onset, but can be recorded as early as 100ms after stimulus onset [31]—or as late as 470ms after the stimulus onset [32]). It is worth investigating early visual activation with other well-established electrophysiological correlates of visual stimuli processing appearing in the same time window, namely N170 and EPN. The overlap between N170 and EPN and VAN is also interesting from a theoretical point of view, as it is questionable whether VAN analyses does indeed produce more information than analyses of classical ERP components that are associated with the early visual processing of emotional stimuli and have not previously been discussed in the context of awareness studies.

Indeed, several studies seem to support the hypothesis that N170 and EPN components are more pronounced when participants are aware of the facial stimuli. For instance, Eimer, Kiss & Holmes (2008) have observed the EPN component for fearful faces both under subliminal and supraliminal conditions. Interestingly, regarding supraliminal stimuli, the ERP component was more pronounced in cases of correct emotion discrimination. This suggests that EPN predicts accuracy of emotional face detection, which in turn is often shown to correlate with stimulus awareness; therefore, one may argue that an increase of accuracy should be associated with increased awareness (see e.g. [33], but see S1 Text, point 1). In the same vein, [19] have shown a more pronounced amplitude of the N170 component for fearful faces. The effect was also observed under exposure to both subliminal and supraliminal stimuli, although it was more pronounced at shorter presentation times. It should be mentioned that the participants’ task in this study was to identify emotion expressions, thus even at the shortest presentation times attention might have been required. Also, this study has utilised stimuli that have been modified so that facial features (eyes, eyebrows, mouths) were more pronounced than the rest of the face. Thus, it is probable that participants in this study were aware of the stimuli even in the subliminal condition. Finally, [20, 25] showed that attention might modulate facial emotion expression processing. Their results indicated that the N170 amplitude is more pronounced when top-down attention is engaged in facial expression judgements. Similar results were observed with the EPN component [20]. Assuming that awareness is closely related to top-down attentional amplification (see e.g. [2]), one might expect that the N170 amplitude could also predict awareness of processing facial expressions.

It is important to note that none of the aforementioned ERP studies explicitly examined participants' awareness. Some of the studies investigated the N170 and EPN components under the subliminal conditions [18–19]. However, it is well known that some participants may be aware of visual stimuli even under 17ms exposures [34]. In the same vein, supraliminal exposure also does not guarantee participants’ awareness of all presented faces, e.g. due to attention fluctuation, fatigue, etc. Thus, a trial-by-trial test of awareness should be introduced to examine awareness of each stimulus. Multiple methods have recently been proposed to fulfil this requirement (see: [33] for the review), such as a perceptual awareness scale that measures stimulus visibility, or confidence ratings and post-decision wagering scales that focus on measuring metacognitive awareness. In our recent study we have applied all three scales in the context of a fearful face identification task [35], with results indicating that they all successfully probed awareness. We also did not observe any systematic differences between the scales that had made us believe that visibility scale measures the very same variable that is probed with post-decision wagering and confidence ratings, namely metacognitive awareness of a stimulus (see also: [36]). The most surprising result in this study was that post-decision wagering (in which certainty in decisions is expressed by betting with imaginary money) biased participants' performance to the extent that enhanced accuracy of emotional face detection was observed. We interpreted this result as an effect of the motivation induced by monetary incentives involved in the wagering task. However, it still remains unknown whether late visual processing (attentional effect) was enhanced by motivation or only by early visual processing of the stimuli.

Here, we aim to address this problem by replicating the effect of enhanced emotional face identification under post-decision wagering task conditions [35]. We aim to investigate how PDW compared to CR affects (1) the identification of emotional faces, (2) the relation between awareness and performance observed, and (3) the neural correlates of awareness. We believe that aside of this methodological goals, comparing the ERP correlates of consciousness for both scales may shed a light on the very discussion on NCCs. Therefore, we test whether the electrophysiological correlates of awareness reported in the previous studies (i.e. P1, ERP components recoded within the VAN time window and P3) are observed with both metacognitive scales of awareness when facial expressions are used as stimuli. We expect the presence of specific ERP components associated with facial emotion expression perception within the VAN time window (i.e. N170 and EPN). Finally, we compare patterns of behavioural results and ERP components when awareness is probed with confidence ratings and post decision wagering. While investigating the differences between results observed with both scales, we discuss both the methodological and theoretical consequences of our results.

## Method

### Participants

Forty-seven Jagiellonian University undergraduate students (17 females, mean age = 21.30, SD = 1.75) voluntarily participated in the study in exchange for course credits. All participants were right-handed with normal or corrected to normal vision and had no neurological or psychiatric history. Participants provided written informed consent before the experiment. The study was approved by the Ethics Committee for Experimental Research at the Institute of Psychology, Jagiellonian University.

### Procedure and design

#### Stimuli and apparatus

Participants were seated in a dimly lit, sound-attenuated and electrically shielded cabin. A computer screen was placed at a viewing distance of 60 cm. The task procedure was displayed on a ViewSonic vx2268wm monitor (screen refresh rate: 100 Hz) controlled by an nVidia GeForce graphics card.

We used a backward masking task in which fearful or neutral facial stimuli were used as targets. A complex pattern formed with the fragments of faces in random positions served as a mask. We chose 20 fearful and 20 neutral faces (equal proportions of male and female faces) from the NimStim set [37]. Each face picture was converted to grey scale. Stimulus size equalled 4° x 5° of the visual angle. All stimuli were presented at the centre of the computer screen. The mean of average luminance (measured in HSL space) of pictures showing fearful faces was 129.17 (SD = 8.15), and was similar to the luminance of neutral faces (131,55 (SD = 8.95)). The mask luminance was 123,75 (SD<0,01).

#### Procedure

We applied a behavioural procedure described below that was similar to those applied in our previous study [35] with three important modifications that were introduced to allow EEG data analyses. Firstly, we changed the mask so an abstract pattern was used instead of a neutral face. This allowed us to identify ERP components of face processing resulting from the target (not mask) presentation (which also seems to be a problem in some of the previous studies. See e.g. [18–19], but also S1 Text, point 2). Secondly, we combined emotion detection and awareness rating responses into one response key in a similar fashion to [38]. The response key simplification responses allowed us to clarify EEG data analyses, resulting in two (aware vs. unaware) but not four awareness conditions. Finally, we switch from the in-between to within subject design in order to account for the differences in the EEG signal between the CR and PDW conditions. In order to avoid the influence of increased arousal expected in the PDW condition on the subsequent performance with the CR, we set the order of the tasks so that CR always precedes PDW.

**Fig 1:**
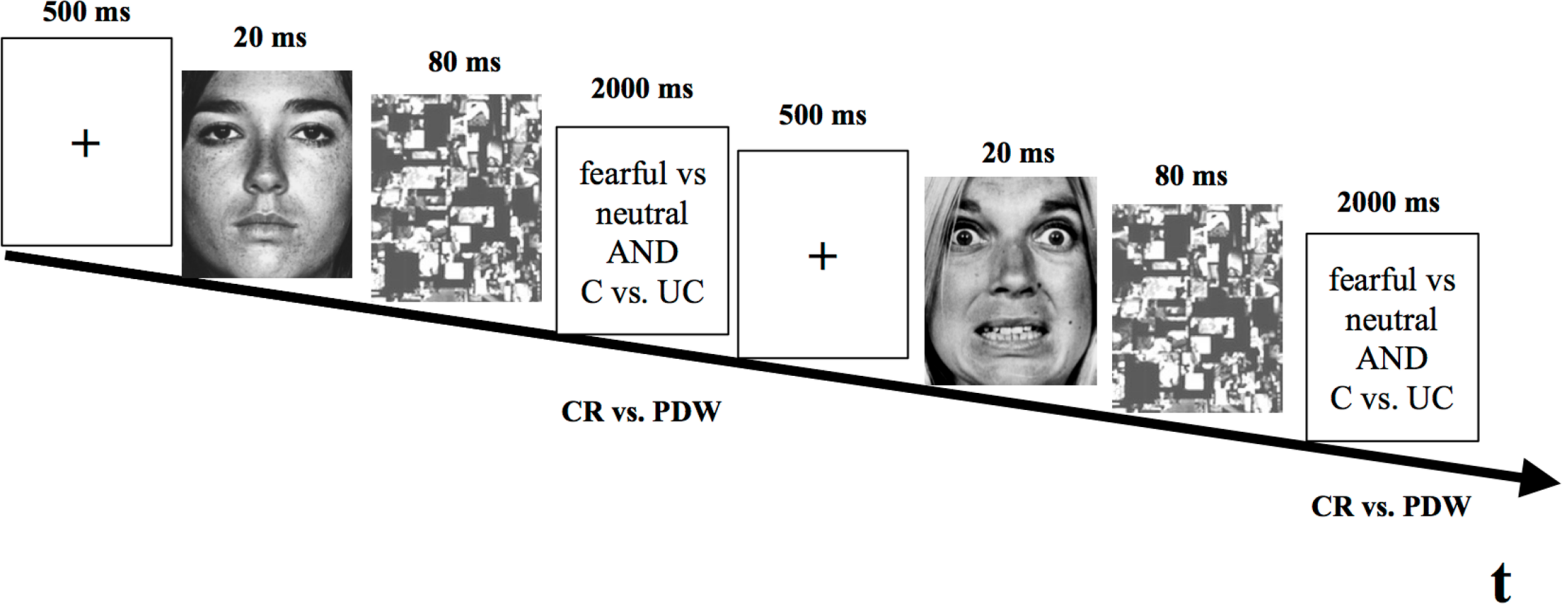
Diagram of experimental procedure of emotional faces identification task with subsequent trials; At each trial a target face (fearful of neutral) was presented, followed immediately by a mask. Then, participants had to discriminate whether a target face was fearful or neutral, and rate their awareness with CR or PDW. Awareness scales were used simultaneously with identification task responses.

Each trial of the procedure consisted of a fixation point (500ms), prime (20ms), mask (280ms) and response key (self-passed with 2 sec time limit). A white cross, presented at the centre of the computer screen (black background), served as a fixation point. Then, a target face (fearful or a neutral) followed immediately by a mask (complex pattern) was displayed at the centre of the computer screen. Finally, participants had to discriminate whether a target face was fearful or neutral, and simultaneously rate their awareness using the confidence ratings or post-decision wagering scales. The awareness scales were applied across separate blocks. No feedback was provided. After a 200ms inter-trial interval a new trial started. The total duration of each trial did not exceed 3s (see Fig 1).

Participants were requested to discriminate facial emotions and rate their awareness of a target facial emotion expression by choosing the key relevant to each possible response (Z -"fearful and aware"; X—"fearful and unaware"; N—"neutral and unaware" and M—"neutral and aware"—see also S1 Appendix). Participants expressed their awareness either with a confidence rating (CR) (unaware = guessing vs. aware = confident) or as wagers with imaginary money, placing a wager of 5 PLN (unaware) or 20 PLN (aware) on face emotion detection judgments (1 PLN equals around 0.25 EUR). Participants gained the wagered amount for correct discriminations and lost that amount for incorrect discriminations. Participants started with a balance of 200 PLN, but no information about gains or losses was provided in the course of the procedure.

Participants performed the masking task in two experimental conditions, divided into separate awareness scale blocks. There were four blocks: two blocks with CR and then two blocks with PDW. Each block contained 60 trials, so the whole procedure consisted of 240 trials. Trials were presented in a random order, with half of trials containing fearful and neutral faces as targets.

#### ERP Recording and Analysis

The EEG was recorded using a BioSemi ActiveTwo system with Ag–AgCl electrodes on 64 monopolar locations according to the extended 10–20 system [39]. Two additional electrodes, the common mode sense (CMS) active electrode and the driven right leg (DRL) passive electrode were respectively used as reference and ground electrodes (www.biosemi.com/faq/cms&drl.htm). All cephalic electrodes were placed on the scalp using the Electro-Cap. The horizontal and vertical EOGs were monitored by 4 additional electrodes, placed above and below the right eye and in the external canthi of both eyes. The EEG was acquired at a sampling rate of 512 Hz. The output data was subsequently transferred to computer and stored for further analyses. The EEG data was filtered off-line with a bandpass of 0.016–45 Hz (24 dB), and sampled for the 650ms trial (150ms prior to the stimulus onset and 500ms after the stimulus onset) using BrainVision software. Trials with various types of artifacts (muscular or blinks) were rejected, with a criterion of ±75 μV. Finally, the data were corrected for eye-movement artefacts [40] and re-referenced to average montage.

Key-press response times were measured for each correct response, and only artefact-free EEG obtained in response to correctly identified stimuli was averaged (for details see S1 Text, point 3). Separate averages were computed for all combinations of task (CR vs. PDW), awareness conditions (aware vs. unaware), and facial expressions (neutral vs. fearful), resulting in eight average waveforms for each electrode and participant. The number of unaware and aware trials depended on participants' ratings. ERP analyses were calculated on correct trials averaged under eight conditions (for details see S1 Text, point 4).

The analyses were performed separately for mean amplitudes of P1, N170, EPN, and P3. These components were defined as the mean voltage within 90-125ms, 145-180ms, 240-360ms, and 350-450ms after the stimulus onset, respectively. These time windows were chosen according to previous studies [20, 26, 30, 41–42]. The amplitudes of these components were calculated relative to the pre-stimulus baseline. Analysis of the effect on the P1 component was performed on three occipital electrodes (O1, Oz, O2), analysis of N170 component was restricted to six lateral occipito-temporal sites (PO7, P7, P9, PO8, P8, P10), while analysis of the EPN component was conducted at three occipital and six occipito-temporal electrodes (O1, Oz, O2, PO7, P7, P9, PO8, P8, P10). Analysis of P3 amplitude was restricted to two parietal sites (CPz and Pz).

Six lateral occipito-temporal electrodes were chosen because N170 has been found to be maximal at these locations [41]. Similarly, electrodes where the EPN component was measured in our study were chosen in line with previous findings [26, 42].

The amplitudes of the P1, N170, EPN, and P3 components were analysed using linear mixed models that were fitted for each ERP component, separately. The models included the subject specific and the electrode specific random intercepts, so we were able to correct for the differences between participants and electrodes, as well as subject specific random rating slopes (Awareness effect). In addition, to account for the variability in voltage estimates resulting from the difference in the number of trials registered for each participant at each condition, we used weighted regression with weights equal to the number of observations that were averaged over to get each data point. The fixed effects chosen to test specific hypotheses include those examining the effects of within-subjects factors of Task (CR vs. PDW), emotional Expression (neutral vs. fearful) and level of Awareness (aware vs. unaware) and their interactions. In addition to the random effects of participants and electrodes, the main model for each ERP component included fixed effects of Task (2), Emotion (2), Awareness (2), and all the possible interactions. The fit of the models was good (χ2 (7) > = 20, p < = .005).

## Behavioural results

The mixed logistic regression models were fitted using the lme4 package in the R Statistical Environment [43–44] using standard (0/1) contrast coding. The main model took into the account fixed effects of Task (2 levels), Expression (2 levels) and Awareness (2 levels), and the interactions between them. Participant specific intercept (Type 2 criterion) and rating slope (Awareness effect) were included as random effects. The model had a good fit (χ2 (9) = 953, p < .001). Statistical significance was assessed by means of the Wald test. Additional models were fitted to compare the signal detection theory parameters between conditions and account for the reaction time effects.

Firstly, we did not observe any difference in facial emotion detection performance between Task conditions. The response criterion for both scales was comparable (*z* = -1.3, *p* > .2). We also compared d' and found no difference (*z* = 1.1, *p* > .28). We also did not observe any difference in response accuracy (*z* = 1.2, *p* > .21). The only difference observed was the main effect of Expression: the criterion was shifted towards fearful faces regardless of the Task condition (*z* = -11.1, *p* < .001). In sum, we did not observe any results suggesting that possible differences between scales are a result of the elevated accuracy in either condition. The facial emotion detection and thus the strength of the perceptual stimulation seem to be comparable between conditions.

**Fig 2:**
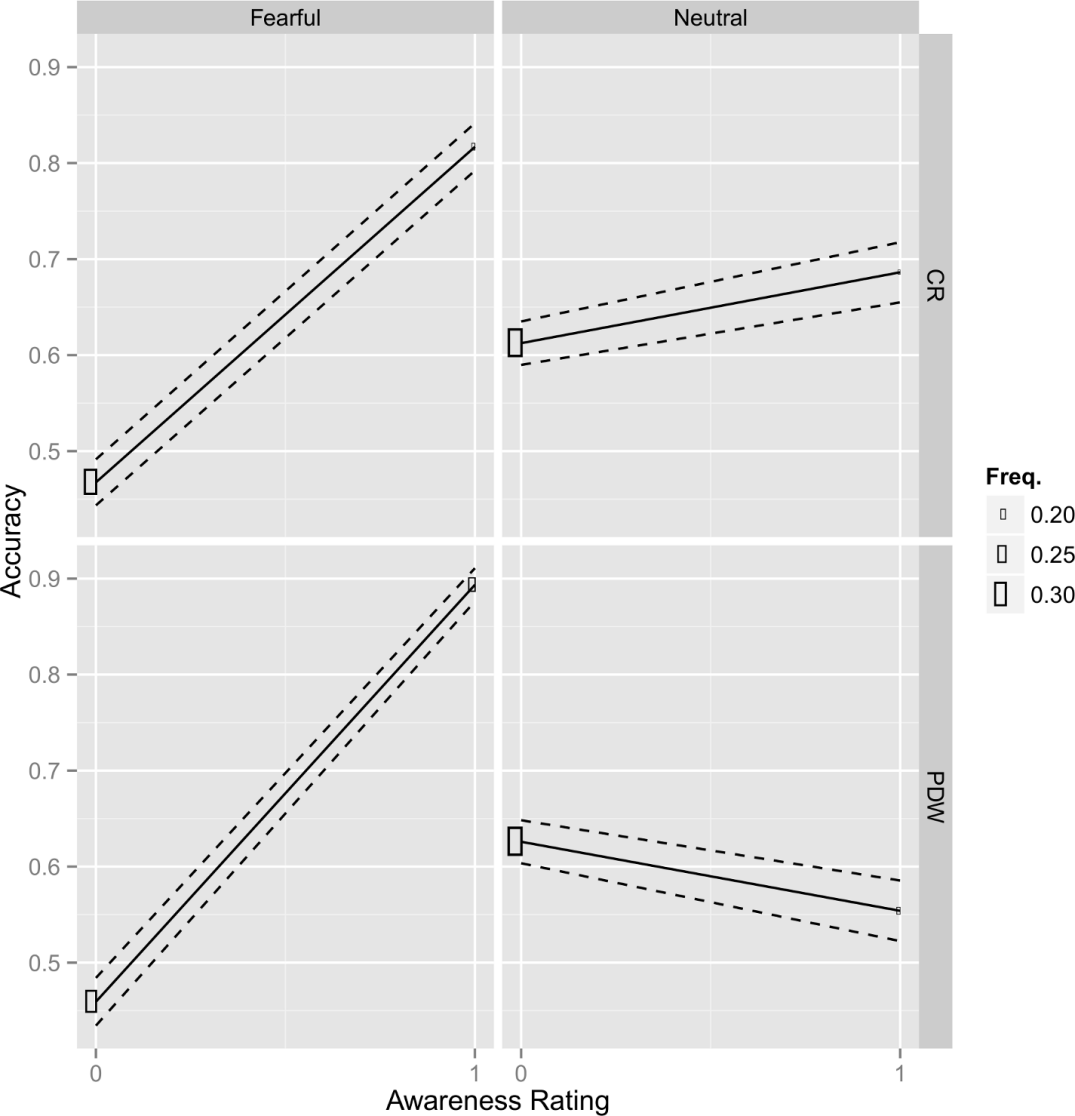
Average accuracy and scale response frequency for the relationship between accuracy and confidence ratings in each condition (CR—confidence ratings; PDW—post decision wagering) and each type of stimuli (F—fearful; N—neutral). Filled circles represent average accuracy for each scale point. The size of the scale points represents the frequency of responses (i.e. proportion of each confidence rating response in each condition).

In the second part of the analysis we compared awareness ratings sensitivity between scales (see also Fig 2). The effect of Awareness was positive and significant for both the fearful and the neutral faces when the CR scale was used (z = 15.2, p < .001; z = 2.8, p < .01 respectively), it was also higher for the fearful than for the neutral faces (z = 9.7, p < .001). However, when the PDW scale was used the effect of Awareness was positive only for the fearful faces (z = 20.2, p < .001) and it was negative for the neutral faces (z = -4.0, p < .001). For the fearful faces the effect of Awareness was higher for the PDW scale than for the CR scale (z = 4.6, p < .001). In short, the results clearly show that even though the facial emotion detection performance was comparable between conditions, participants were more aware of fearful stimuli, especially when the awareness was measured with PDW scale.

Finally, we also ran additional analyses to estimate the effects of reaction times that may provide an alternative explanation for our results. The reaction times were shorter in PDW scales for both fearful and neutral stimuli (*z* ≥ 17.3, *p* < .001). However, when the reaction time was added to our main mixed regression model as an additional fixed effect, even though the fit was improved (χ2(1) = 13.9, *p* < .001) the pattern of results was the same. The effect of Awareness was still positive and significant for both the fearful and the neutral faces when the CR scale was used (z = 9.99, p < .001; z = 2.07, p < .05 respectively) and it was higher for the fearful than for the neutral faces (z = 9.51, p < .001). However, when the PDW scale was used, the effect of Awareness was positive only for the fearful faces (z = 13.9, p < .001) and was negative for the neutral faces (z = -1.94, p = .052). For the fearful faces the effect of Awareness was higher for the PDW scale than for the CR scale (z = 4.65, p < .001).

To sum up, it seems that there are two independent alternative explanations that could be used to explain differences in the regression slopes, i.e. difference in the task performance (accuracy, d', time/accuracy trade-off) and the difference in the Type 2 criterion. The former was excluded by the analyses presented above. The latter seems to be excluded by the very fact that we have used a logistic transformation that makes Type 2 criterion (intercept) and Awareness slope independent.

## Electrophysiological Results

**Fig 3:**
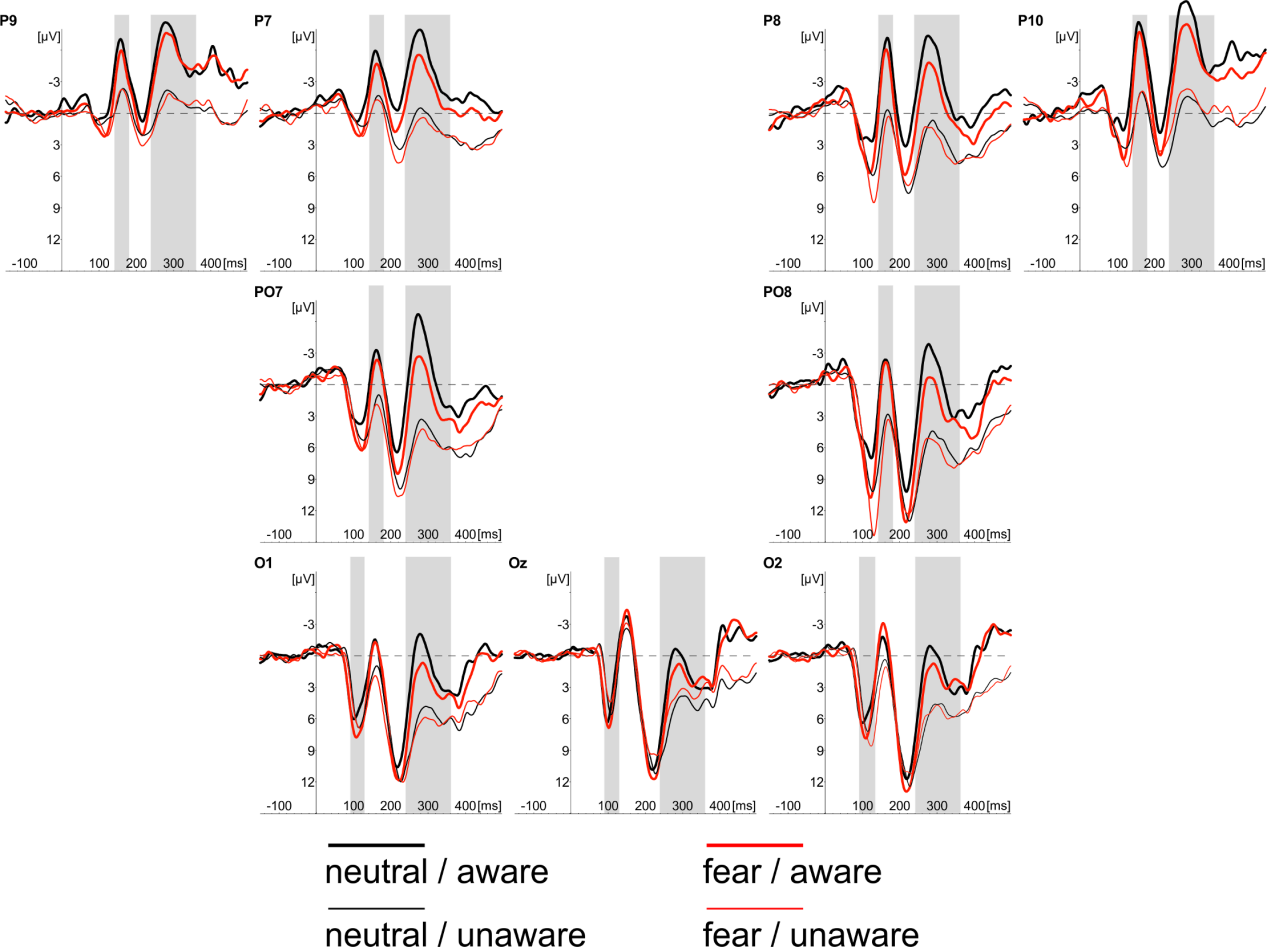
Grand average of ERPs recorded in CR task at occipital (O1, Oz, O2) and occipito-temporal (P7, PO7, P9, P8, PO8, P10) electrodes in response to neutral (black lines) and fearful faces (red lines). Responses obtained in “unaware” (thin lines) and “aware” trails (thick lines) are also shown. Time windows for the region of interest (P1 component; 90-115ms post-stimulus; N170 component; 145-180ms post-stimulus; EPN component 240–340 ms post-stimulus) are highlighted at corresponding electrodes; time 0 indicates stimulus onset.

**Fig 4:**
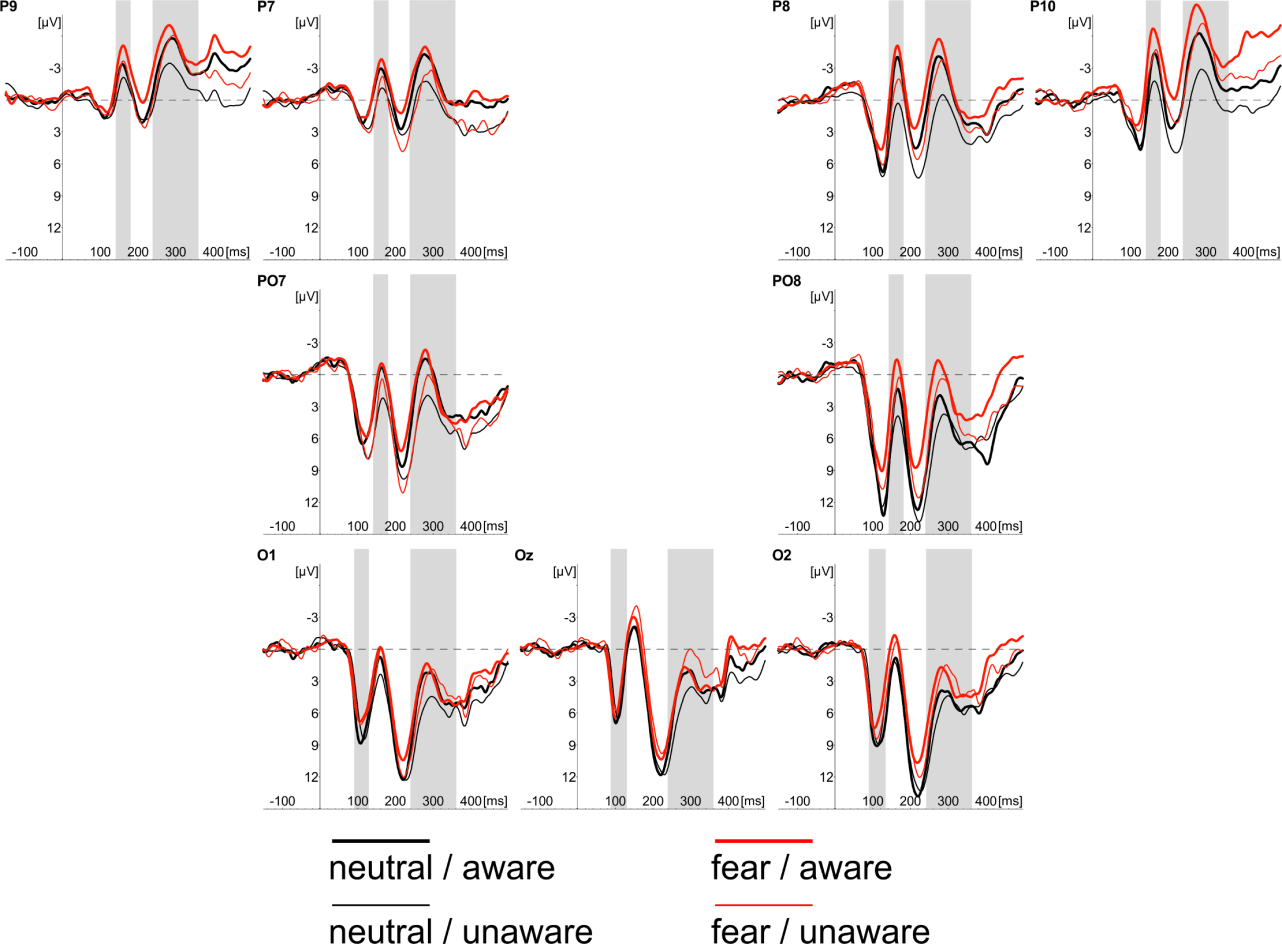
Grand average of ERPs recorded in PDW task at occipital (O1, Oz, O2) and occipito-temporal (P7, PO7, P9, P8, PO8, P10) electrodes in response to neutral (black lines) and fearful faces (red lines). Responses obtained in “unaware” (thin lines) and “aware” trails (thick lines) are also shown. Time windows for the region of interest (P1 component; 90-115ms post-stimulus; N170 component; 145-180ms post-stimulus; EPN component 240–340 ms post-stimulus) are highlighted at corresponding electrodes; time 0 indicates stimulus onset.

**Fig 5:**
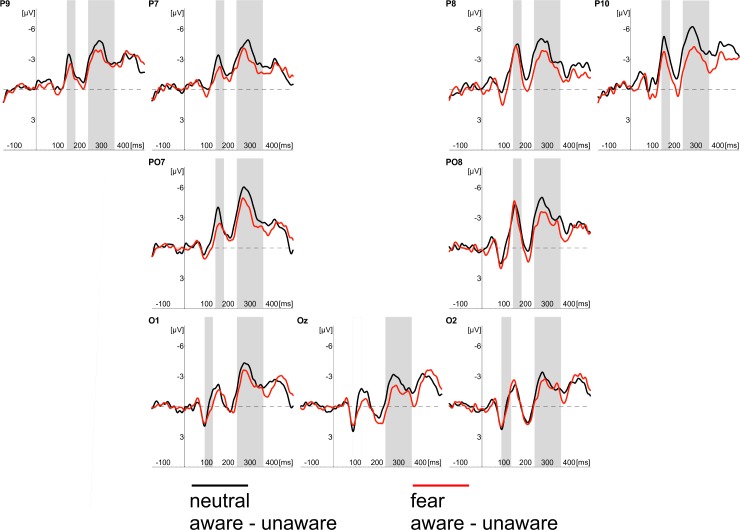
Difference waves computed as differences between aware and unaware conditions recorded in the CR task at occipital (O1, Oz, O2) and occipito-temporal (P7, PO7, P9, P8, PO8, P10) electrodes in response to neutral (black lines) and fearful faces (red lines). Time windows for the region of interest (P1 component; 90-115ms post-stimulus; N170 component; 145-180ms post-stimulus; EPN component 240–340 ms post-stimulus) are highlighted at corresponding electrodes; time 0 indicates stimulus onset.

**Fig 6:**
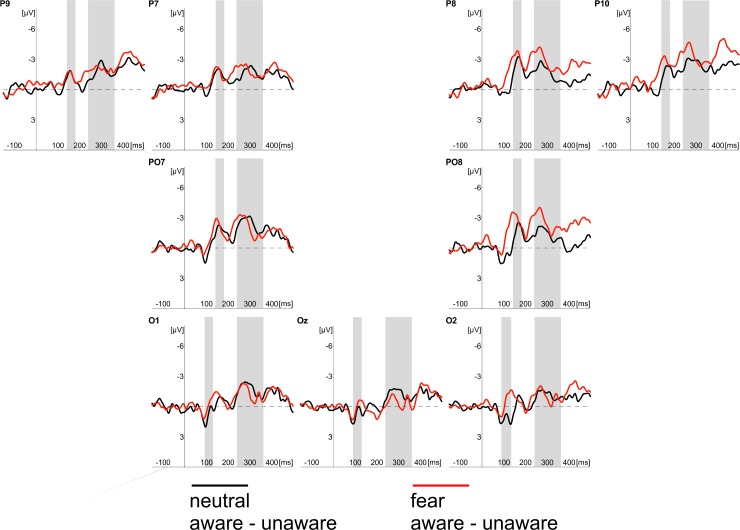
Difference waves computed as differences between aware and unaware conditions recorded in the PDW task at occipital (O1, Oz, O2) and occipito-temporal (P7, PO7, P9, P8, PO8, P10) electrodes in response to neutral (black lines) and fearful faces (red lines). Time windows for the region of interest (P1 component; 90-115ms post-stimulus; N170 component; 145-180ms post-stimulus; EPN component 240–340 ms post-stimulus) are highlighted at corresponding electrodes; time 0 indicates stimulus onset.

**Fig 7:**
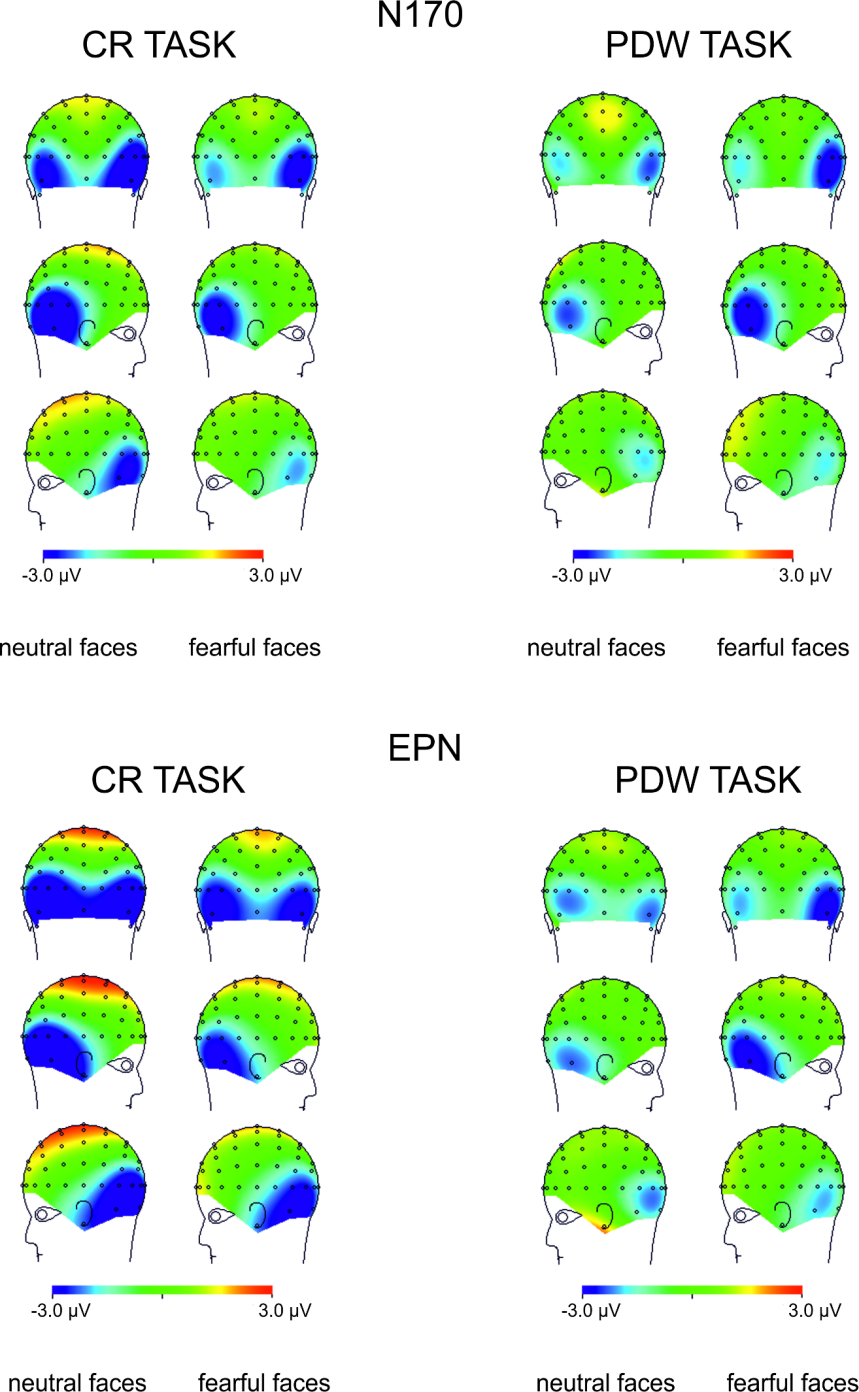
Topographical maps representing voltage differences between brain activity recorded in “aware” and “unaware” trials in the N170 and EPN components time interval (145-180ms post-stimulus and 240-340ms post-stimulus, respectively) illustrated separately for responses to neutral and fearful faces.

We noticed consistent effects of stimulus awareness in both tasks. ERPs recorded in “aware” trials started to differ from ERPs measured in “unaware” trials at 145ms after stimulus onset at the occipito-temporal locations. This effect was evident in CR and PDW tasks. Similar effects of awareness were also evident between 240 and 360ms after stimulus onset at occipotal and occipito-temporal locations (see Figs 3–7).

**Fig 8:**
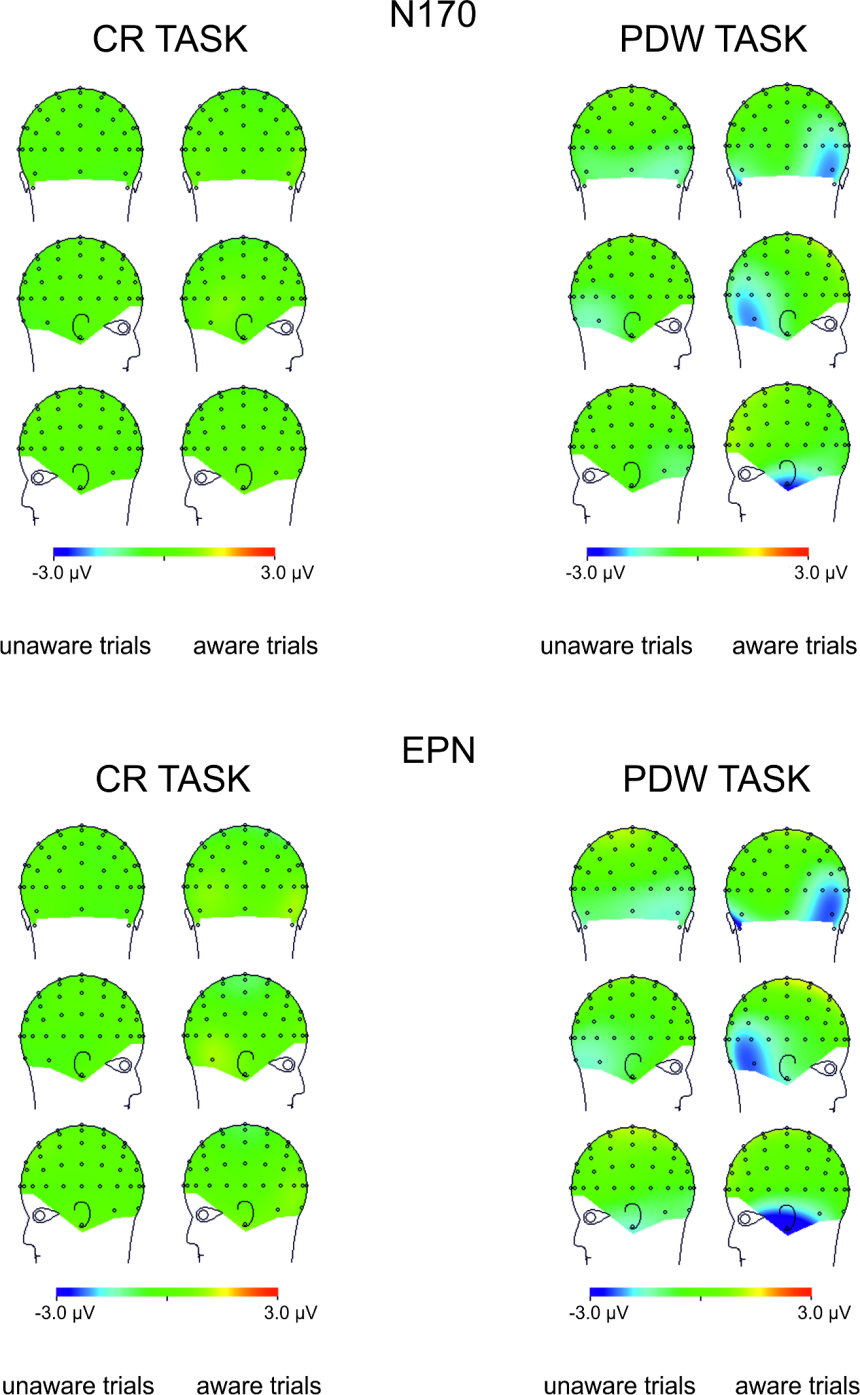
Topographical maps representing voltage differences between brain activity recorded in response to fearful and neutral faces in the N170 and EPN components time interval (145-180ms post-stimulus and 240-340ms post-stimulus, respectively) illustrated separately for “aware” and “unaware” trials.

The inspection of Fig 8 shows that the effect of the emotional expression was virtually absent in the CR task. In contrast, the enhanced negativity (started 145ms post-stimulus) elicited by consciously detected fearful faces, as compared to consciously perceived neutral faces, was observed in the PDW task. A similar pattern of results was observed for the latency window of 240-360ms after stimulus onset for the EPN component. Again, the effect of emotional expression was absent in the CR task. However, ERPs elicited by fearful faces differed from ERPs measured in response to neutral stimuli in the PDW task. The enhanced negativity was obtained in response to the emotional stimuli.

**Fig 9:**
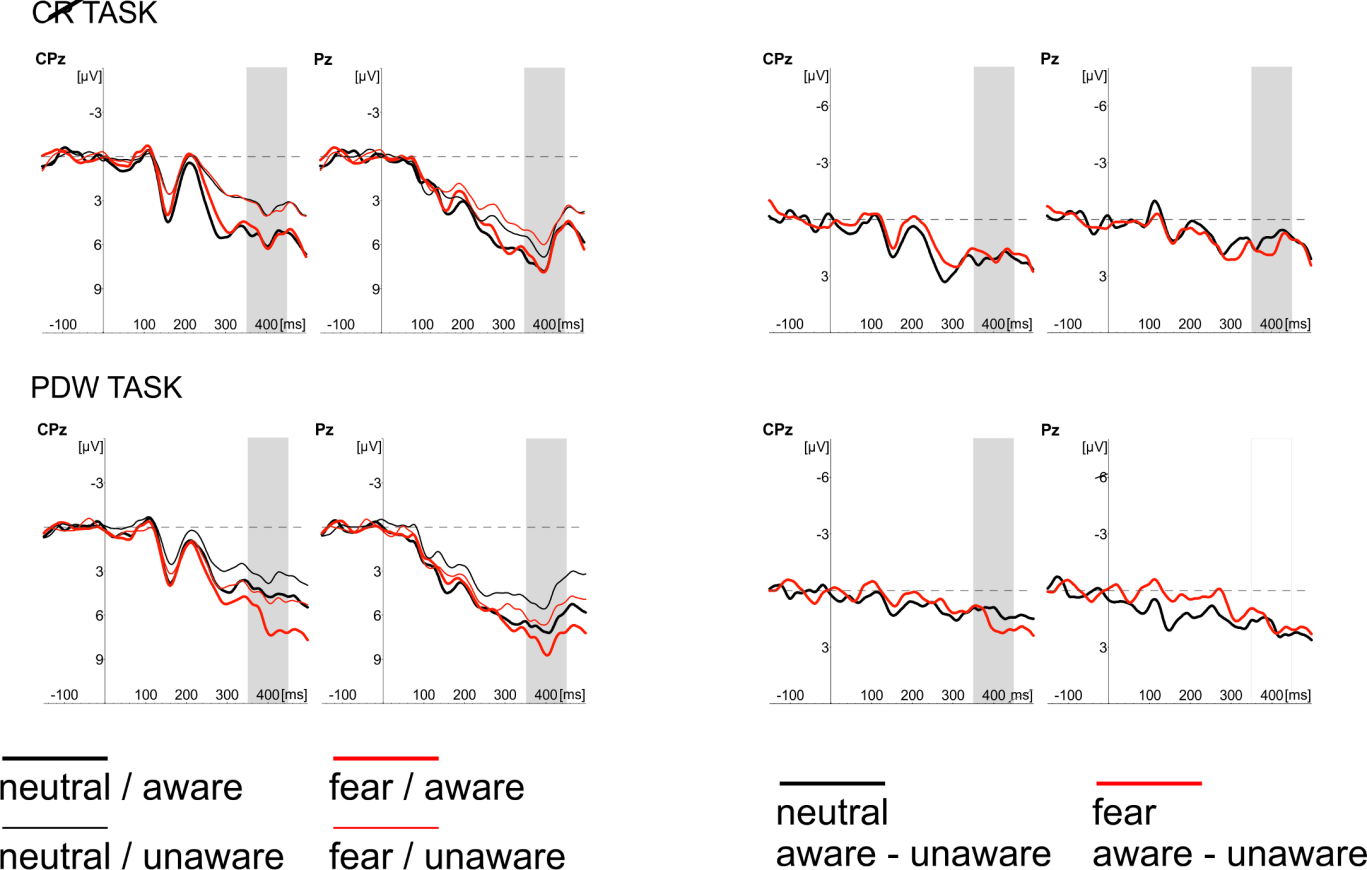
Grand average of ERPs recorded in CR and PDW tasks at parietal electrodes (CPz, Pz) in response to neutral (black lines) and fearful faces (red lines)–left panel. Responses obtained in “unaware” (thin lines) and “aware” trails (thick lines) are also shown. The right panel shows difference waves computed as a difference between aware and unaware conditions recorded in CR and PDW tasks at parietal electrodes (CPz, Pz) in response to neutral (black lines) and fearful faces (red lines). Time windows for the region of interest (P3 component; 300–400 ms post-stimulus) are highlighted at corresponding electrodes; time 0 indicates stimulus onset.

**Fig 10:**
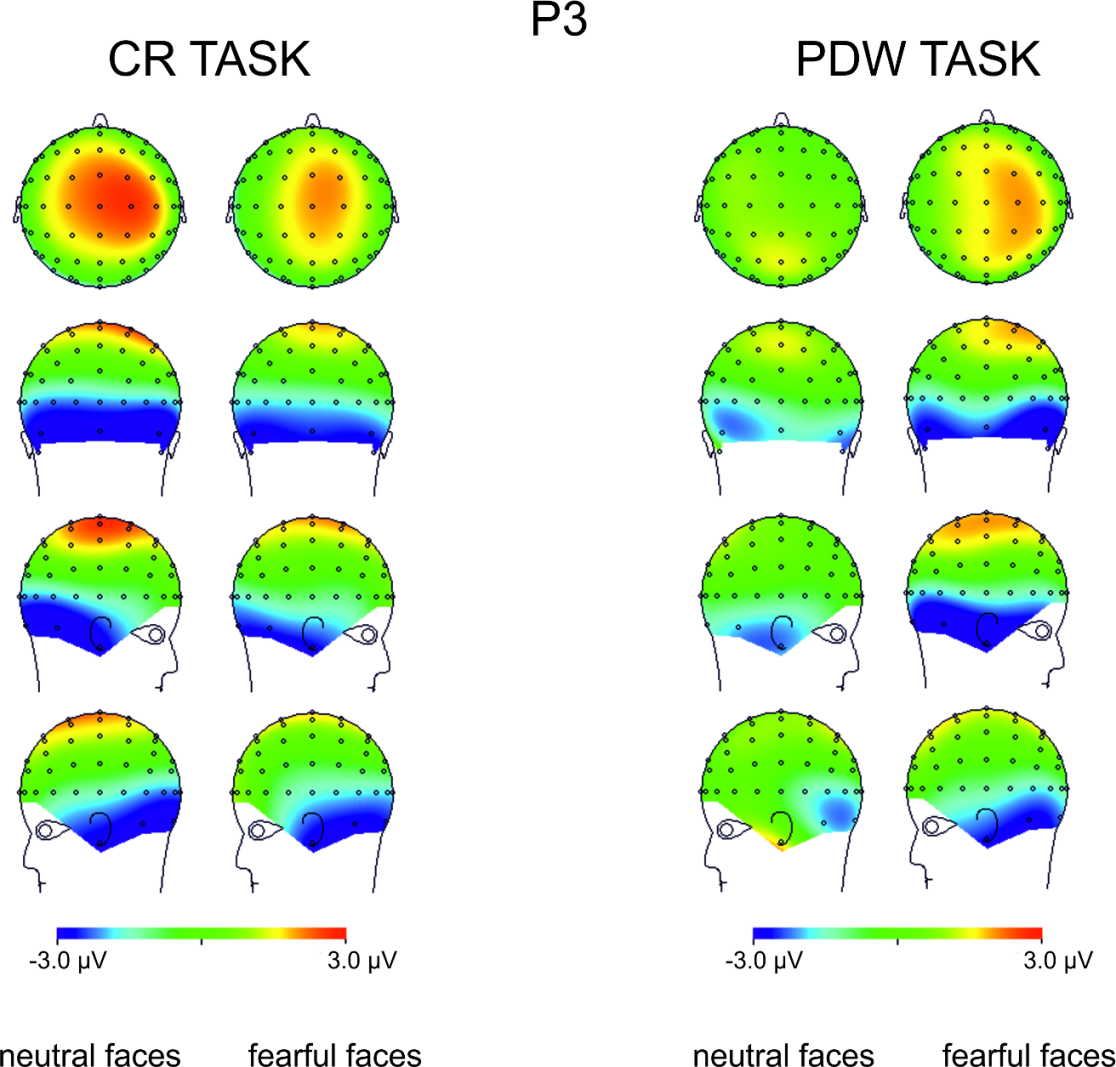
Topographical maps representing voltage differences between brain activity recorded in “aware” and “unaware” trials in the P3 component time interval (300-400ms post-stimulus) illustrated separately for responses to neutral and fearful faces.

Finally, the effect of awareness was also evident at centro-parietal sites between 350 and 450ms after stimulus onset (see Figs 9 and 10). Here, the more pronounced amplitude of P3 components was observed for "aware" trials. Similar pattern was found for the emotional stimuli. The amplitudes of N170, EPN and P3 components were more pronounced when fearful faces were presented. Interestingly, the main effect of Task was observed only in the P1 latency window, where PDW amplitude was more pronounced. Those findings were confirmed by the further statistical analyses presented in the next subsections.

### P1 Component

The amplitudes of the P1 component were compared with a fitted linear mixed model specified above. Following our hypotheses, we analysed the main effect of Awareness and Task, as well as the main effect of Expression and their interactions. The main effects of Awareness and Expression were not significant (*t*(912) = .89, *ns* and *t*(959) = -1.44, *ns*, respectively). We observed the significant main effect of Task (*t*(959) = 2.80, *p* < .05). None of the two-way interactions proved to be significant (*t's*(>900) < 1.4, *ns*). The three-way interaction (i.e. Awareness x Expression x Task) was also not significant (*t*(906) = - .99, *ns*)

To sum up, the only effect observed with the P1 component is the difference between CR and PDW suggesting that that early stages of stimulus processing differ depending on the scale applied (amplitude was more pronounced under PDW). This result suggests that early phases of stimulus processing differ depending on the type of the task participants are involved in.

### N170 component

The similar schema of analyses using fitted linear mixed model was applied to amplitudes of the N170 component. Again, following our hypotheses, we analysed the main effect of Awareness and Task, as well as the main effect of Expression and their interactions. The main effects of Awareness was significant (*t*(1920) = -6.61, *p* < .001). We also observed the significant effect of Expression (*t*(1967) = -3.80, *p* < .001). Those differences are illustrated in Figs 3 and 4, which show ERP responses to “aware” and “unaware” trials at occipito-temporal electrodes (PO7, P7, P9, P8, PO8, P10) separately for each task and each expression and, even more clearly, in Figs 7 and 8 showing Awareness and Expression effects with spline maps. The main effect of Task was not significant (*t*(1967) = 0.56, *ns*). Again, none of the two-way interactions proved to be significant (*t's*(>1900) < 1.52, *ns*). The three-way interaction (i.e. Awareness x Expression x Task) was also not significant (*t*(1914) = - 1.22, *ns*)

To sum up, the amplitude of N170 was more pronounced when participants were aware of stimuli and when the stimuli were emotional.

### EPN component

Fitted linear mixed model was also applied to compare amplitudes for the latency window of EPN component. The main effects of Awareness and Task, as well as the main effect of Expression and their interactions were again analysed. The main effect of Awareness was significant (*t*(2928) = -6.70, *p* < .001). We also observed the significant main effect of Expression (*t*(2975) = -4.89, *p* < .001) and the significant main effect of Task (*t*(2975) = -3.79, *p* < .001). We also observed a significant interaction between Awareness and Task (*t*(2926) = 3.78, *p* < .001) and Expression and Task (*t*(2973) = -2.28, *p* < .05). The interaction between Expression and Awareness was not significant (*t*(2926) *= —*.*53*, *ns*). The three-way interaction (i.e. Awareness x Expression x Task) was also not significant (*t*(2922) = -.07, *ns*).

All the differences are illustrated in Figs 3 and 4, which show ERP responses for all conditions and in Figs 7 and 8 showing Awareness and Expression effects with spline maps.

To sum up, the amplitude of EPN was more pronounced when participants were aware of stimuli, when the stimuli were emotional and when using PDW scale. Interactions suggest that for PDW scale enhanced negativity was observed at both aware and emotional trials.

### The P3 component

Finally, fitted linear mixed model was also applied to compare amplitudes for the latency window of P3 component. The main effects of Awareness and Task, as well as the main effect of Expression and their interactions were again analysed. The main effects of Awareness was significant (*t*(576) = 4.07, *p* < .001). We also observed the significant main effect of Expression (*t*(623) = 4.22, *p* < .001) and the significant main effect of Task (*t*(623) = 4.09, *p* < .001). We also again observed the significant interaction between Awareness and Task (*t*(574) = 2.71, *p* < .01) and Expression and Task (*t*(621) = 4.87, *p* < .001). The interaction between Expression and Awareness was not significant (*t*(574) *=* .49, *ns*). The three-way interaction (i.e. Awareness x Expression x Task) was also not significant (*t*(570) = -.51, *ns*).

All the differences are illustrated in Fig 9, which show ERP responses for all conditions and in Fig 10 showing Awareness effect with spline maps.

To sum up, the amplitude of P3 was more pronounced when participants were aware of stimuli, when the stimuli were emotional and when using PDW scale. Interactions suggest that for PDW scale enhanced negativity was observed at both aware and emotional trials.

## Discussion

The present study investigated electrophysiological correlates of awareness by examining ERP components registered under the backward masking task. The awareness was probed with confidence ratings and post-decision wagering scales. Our behavioural analysis shows that both scales are equally sensitive to the difference in emotion identification task accuracy in aware compared to unaware conditions. The ERP analysis clearly shows that awareness under both CR and PDW tasks is related to the more pronounced amplitudes of the N170, EPN, and P3 components. Thus, our study indicates that awareness ratings are predicted by ERP components that are associated with enhanced stimuli identification at early stages of information processing (i.e. N170 and EPN), but also by the P3 component that is often associated with attention and controlled processing. However, there is an important question concerning whether all ERP components measured in this study are indeed the NCC of awareness?

Apparently, our results are consistent with those observed by [8, 31], who suggested that VAN correlates with the conscious perception. Indeed, we observed an enhanced negativity within the VAN window that correlates with the awareness ratings expressed with confidence. However, it is worth noting that the N170 and EPN components (claimed to be specific to the processing of emotional facial expressions) were observed precisely within the VAN time window (see Figs 4 and 5). This raises the question of whether enhanced negativity observed at the VAN window is a neural correlate of awareness or whether it reflects the better processing of facial stimuli that only later result with increased awareness. This is important, as both N170 and EPN are observed even when participants are not aware of stimuli (in both our studies and previous studies [18–19, 30]). One may argue that an overlap of VAN and N170 and EPN components is not problematic, as VAN should occur over the ERP components (i.e. that negativity is even more enhanced than the negativity which is a result of the face processing components). However, it also may be suggested that the more pronounced N170 and EPN components reflect a more detailed, sensitive and focussed processing of facial stimulus that only at later stages of processing result in higher awareness [45–46]. Thus, N170 and EPN would be necessary but not sufficient correlates of awareness. This is also very likely because we used confidence ratings and post-decision wagering ratings that probe participants’ judgments on visual awareness. Such judgments are instead based on the quality of earlier visual processing. Consequently, we believe that the relation between typical ERP components observed within the VAN window and VAN itself are worth investigating. For this purpose, one may compare the VAN and various early visual ERP components associated with different types of visual stimuli processing. This could be examined by comparing facial stimuli processing components as proposed within this study with other ERP components of the processing of specific content (e.g. N2pc in cases of lateral presentation of stimuli). It will be interesting to see whether all the early visual components will be related to the awareness ratings and whether VAN will be observed within the same time window for different types of stimuli. We believe that such studies may point towards an alternative interpretation for VAN in which VAN reflects brain activity that is actually related to processing of specific visual content, but not necessarily awareness.

Finally, we also observed enhanced P3 amplitude for aware trials. This finding is consistent with many previous reports (see [8] for the review). At the same time, such results should be treated with caution, as it is not clear whether the amplitude of P3 is a solid candidate for an electrophysiological correlate of awareness. The elevated P3 component can be obtained as a response to rare targets even when stimuli are presented subliminally and perceived unconsciously (e.g. oddball task—see: [47]). It is worth noting that the P3 component was clearly visible in unaware trials in this and many previous studies (see e.g. [8]). A recent study also suggests that P3 is related to task-relevant processing rather than awareness (see: [48]). In the same vein, P3 is absent or very small in such paradigms when elicited by standard stimuli requiring no participant’s response [49–51]. To sum up, it is not clear how awareness can be linked to P3 amplitude, since unconsciously processed stimuli are capable of clearly eliciting a visible P3 wave, while other consciously perceived stimuli elicit no P3 at all. Interestingly, this critique is analogous to our doubts on whether N170 and EPN should be treated as NCC. This is because both the early and late visual components may be related to awareness or the processing that precedes (early component) or follows (late components) awareness [45].

The reported study also indicates elevated awareness in facial emotion identification under PDW conditions. The awareness ratings of fearful facial expression identification were more pronounced when participants rated their awareness with the PDW scale. The ERP analyses of N170, EPN and P3 amplitudes confirm the difference in emotional stimuli processing between the PDW and CR conditions indicated with behavioural measures. We propose that these results may also add a new interpretation of the effects reported in our previous studies[35]. For the CR scale, there was no specific effect of fearful stimuli presentation on the N170, EPN and P3 amplitudes. All components were more pronounced when participants were aware of the stimuli. The results were different when the PDW scale was applied. It appeared that the PDW condition led to more efficient identification of emotional stimuli. This effect was clearly visible, especially with EPN (and to some extent P3). It seems that the ERP components are more pronounced for the aware condition in case of fearful stimuli presentations than they were in the unconscious condition and in case of neutral stimuli. Also, when the N170, EPN and P3 components' amplitude voltage differences were directly contrasted for fearful and neutral faces with topographical maps, there was no effect regarding the CR condition, whereas for the PDW condition there was a difference in activation for emotional faces in the time windows of all the components. Taken together, our results suggest that the use of PDW task affects visual processing especially when fearful faces are presented. Therefore, it seems possible that wagering behaviour amplifies processing of emotional stimuli under brief presentation conditions due to stimuli importance that in turn may affect participants' motivation (see e.g.: [52]). This is because fearful faces are important from an evolutionary perspective and are highly relevant to social interactions (see e.g.: [52–53]). Our interpretation is also consistent with results suggesting that fearful stimuli recruit more attentional resources (see: [54]), and their detection occurs earlier than for neutral stimuli [46, 55]. This, in turn, again suggests that all of the ERP components analysed within the study are not necessarily NNC, but they instead reflect higher sensitivity of the system to important stimuli that only later could give rise to awareness.

Since we have observed a difference in the reaction times between the Task conditions, in principle our effects could to some extent be explained by the differences in the task performance: when accuracy is similar, the shorter RTs may indicate that the shorter condition is easier. However, this does not explain the effect of the Scale of Awareness as they were still observed even when we controlled for the RTs in the model.

The main limitation of our study was the use of bimodal awareness scales. We decided to apply the scales in this fashion since there was a risk that an insufficient number of trials at each scale point would be collected to compare ERP components. However, it is also well known that bimodal measures also strongly assume that the scales are sensitive, i.e. they are able to dissociate between awareness and unawareness, but scale ratings might differ in certainty even when one is aware of a visual stimulus (e.g. [36, 56]), e.g. because of the influences of risk aversion (as in case of PDW—see: [57–58]). An interesting issue concerns whether the same correlates will be observed with scales of awareness that simply measure visibility instead of asking about the certainty of the identification decision (e.g. PAS or visual analogue scale—see: [59]). In other words, it is worth examining whether the same pattern of ERPs for emotional stimuli will be observed when using measures that are not related to metacognitive judgement of the identification decision.

To sum up, our study provides substantial evidence indicating that monetary wagering induces elevated sensitivity to emotional stimuli. We identified two early visual ERP components that were observed when participants rated themselves as aware of the stimuli, i.e. N170 and EPN. Both ERP components are functionally related to face detection processing. We also observed more pronounced P3 amplitude for aware conditions. We also indicated more pronounced amplitudes of both EPN and P3 components in the PDW condition in cases of fearful faces. Based on these findings we argue that PDW can potentially amplify attention when detecting important stimuli (such us fearful faces) due to increased motivation induced by monetary incentives. We provided in-depth discussion of the relation between these two correlates of face processing (i.e. N170 and EPN) and the VAN component that is usually taken as an early ERP correlate of awareness. We propose that the time window overlap between the VAN and N170/EPN should be further analysed to clarify whether the enhanced negativity in the VAN window is the result of awareness, or whether it is more detailed processing of stimuli that only later results in metacognitive awareness.

## Supporting Information

S1 Appendix Study Instructions. (DOCX)

S1 Text Annotations. (DOCX)
