# Supplementary material for: Post-Decision Wagering Affects Metacognitive Awareness of Emotional Stimuli: An Event Related Potential Study
Source: PLoS One. 2016 Aug 4;11(8):e0159516. doi: 10.1371/journal.pone.0159516 (PMC4973871; doi:10.1371/journal.pone.0159516)
Supplement: S1 Appendix — (DOCX) [file pone.0159516.s001.docx]

Study instructions

Over the course of this study faces will be presented on the monitor screen. The faces will express one of two emotional states and will be presented in such a way that the recognition will not be easy. Your task is to provide your response with the computer keyboard.

CR condition:

If you are fully confident the face was neutral, press "Z" with the left hand

If you are not confident, but you think the face was neutral, press "X" with the left hand

If you are not confident, but you think the face was fearful, press "N" with the right hand

If you are fully confident, that the face was fearful, press "M" with the right hand

press SPACE to see the graphical version of the scale

| Z | X | N | M |
| --- | --- | --- | --- |
| I am fully confident that NEUTRAL | no confident, but rather NEUTRAL | no confident, but rather FEAR | I am fully confident that FEAR |

PDW condition:

If this was a NEUTRAL face and you bet 20 PLN, press "Z" with the left hand

If you think it was a NEUTRAL face and you bet 5 PLN, press "X" with the left hand

If you think it was a FEARFUL face and you bet 5 PLN, press "N" with the right hand

If this was a FEARFUL face and you bet 20 PLN, press "M" with the right hand

press SPACE to see the graphical version of the scale

| Z | X | N | M |
| --- | --- | --- | --- |
| 20 PLN  NEUTRAL | 5 PLN  NEUTRAL | 5 PLN  FEAR | 20 PLN  FEAR |
