# Supplementary material for: Post-Decision Wagering Affects Metacognitive Awareness of Emotional Stimuli: An Event Related Potential Study
Source: PLoS One. 2016 Aug 4;11(8):e0159516. doi: 10.1371/journal.pone.0159516 (PMC4973871; doi:10.1371/journal.pone.0159516)
Supplement: S1 Text — (DOCX) [file pone.0159516.s002.docx]

Annotations:

. Task performance accuracy often correlates with awareness (see e.g. [33]). It was also sometimes directly used to measure awareness ([60] – so-called objective measure of awareness). However, recently it has often been argued that awareness and performance should not be equated (see e.g. [38, 61]).

2. it is worth noting that neutral face is not only problematic as a mask because of ERP components identification, but also because the activation for neutral faces presented as them asks can contribute to the activation for neutral faces presented as targets, and thus activation for neutral faces is always stronger than those registered with emotional targets (see [19]).

3. The removals were really rare. The average number of removed trials per condition was always lower than one. Only for two participants, we have excluded more than 10 cases in one of the condition

4. In 95 out of 384 data points (participants times condition cells) the number of trials was smaller than 10. The data points with low number of observations were similarly distributed between aware and unaware conditions (55 to 42 respectively) and between fearful and neutral conditions (54 to 43 respectively). Due to the data structure we have applied model of regression that is weighting the data points depending on the number of observations as described above)

Additional References:

1. Vermeiren A, Cleeremans A. The validity of d′ measures. PloS one. 2012 Feb 20;7(2):e31595. doi:10.1371/journal.pone.003159
2. Fleming SM, Weil RS, Nagy Z, Dolan RJ, Rees G. Relating introspective accuracy to individual differences in brain structure. Science. 2010 Sep 17;329(5998):1541-3.
